# Temporal Dynamics of the Microbial Community Composition with a Focus on Toxic Cyanobacteria and Toxin Presence during Harmful Algal Blooms in Two South German Lakes

**DOI:** 10.3389/fmicb.2017.02387

**Published:** 2017-12-04

**Authors:** Pia I. Scherer, Andrew D. Millard, Andreas Miller, Renate Schoen, Uta Raeder, Juergen Geist, Katrin Zwirglmaier

**Affiliations:** ^1^Limnological Research Station Iffeldorf, Aquatic Systems Biology Unit, Department of Life Sciences Weihenstephan, Technical University of Munich, Munich, Germany; ^2^Warwick Medical School, University of Warwick, Coventry, United Kingdom; ^3^Bavarian Health and Food Safety Authority, Oberschleißheim, Germany

**Keywords:** harmful algal bloom, high-throughput sequencing, cyanobacteria, bacterioplankton, microcystin, *Microcystis*, *mcyB*, *mcyE*

## Abstract

Bacterioplankton plays an essential role in aquatic ecosystems, and cyanobacteria are an influential part of the microbiome in many water bodies. In freshwaters used for recreational activities or drinking water, toxic cyanobacteria cause concerns due to the risk of intoxication with cyanotoxins, such as microcystins. In this study, we aimed to unmask relationships between toxicity, cyanobacterial community composition, and environmental factors. At the same time, we assessed the correlation of a genetic marker with microcystin concentration and aimed to identify the main microcystin producer. We used Illumina MiSeq sequencing to study the bacterioplankton in two recreational lakes in South Germany. We quantified a microcystin biosynthesis gene (*mcyB*) using qPCR and linked this information with microcystin concentration to assess toxicity. Microcystin biosynthesis gene (*mcyE*)-clone libraries were used to determine the origin of microcystin biosynthesis genes. Bloom toxicity did not alter the bacterial community composition, which was highly dynamic at the lowest taxonomic level for some phyla such as Cyanobacteria. At the OTU level, we found distinctly different degrees of temporal variation between major bacteria phyla. Cyanobacteria and Bacteroidetes showed drastic temporal changes in their community compositions, while the composition of Actinobacteria remained rather stable in both lakes. The bacterial community composition of Alpha- and Beta-proteobacteria remained stable over time in Lake Klostersee, but it showed temporal variations in Lake Bergknappweiher. The presence of potential microcystin degraders and potential algicidal bacteria amongst prevalent Bacteroidetes and Alphaproteobacteria implied a role of those co-occurring heterotrophic bacteria in cyanobacterial bloom dynamics. Comparison of both lakes studied revealed a large shared microbiome, which was shaped toward the lake specific community composition by environmental factors. Microcystin variants detected were microcystin-LR, -RR, and -YR. The maximum microcystin concentrations measured was 6.7 μg/L, a value still acceptable for recreational waters but not drinking water. Microcystin concentration correlated positively with total phosphorus and *mcyB* copy number. We identified low abundant *Microcystis* sp. as the only microcystin producer in both lakes. Therefore, risk assessment efforts need to take into account the fact that non-dominant species may cause toxicity of the blooms observed.

## Introduction

Cyanobacteria are of special interest in aquatic microbiology. They occur alongside other prokaryotes in aquatic environments and the extent of the interspecies interaction with those bacteria is only just emerging (Li et al., 2011, 2012; Lee et al., 2016; Parulekar et al., 2017). In particular, cyanobacteria capable of producing the hepatotoxic secondary metabolite microcystin cause problems in drinking and recreational water reservoirs (Hudnell, 2010; Roegner et al., 2014; He et al., 2016). For this reason, identification of potentially microcystin-producing species by microscopy and microcystin analyses is commonly used to assess risks associated with toxic cyanobacteria (Chorus and Bartram, 1999; Sangolkar et al., 2006; Kaushik and Balasubramanian, 2013; Gaget et al., 2017).

Deducing the toxicity of an algal bloom based on the cyanobacterial community composition can be difficult because microcystins are potentially produced by different species (Chorus and Bartram, 1999). To address this problem, quantitative polymerase chain reaction (qPCR) assays have been introduced to quantify toxic genotypes (Ostermaier and Kurmayer, 2010; Martins et al., 2011; Al-Tebrineh et al., 2012).

When assessing the microbial diversity in water bodies, culture-independent techniques are commonly used. Molecular approaches outperform microscopy when estimating the diversity of heterotrophic prokaryotes and are increasingly applied to evaluate cyanobacterial community composition. The most widespread molecular methods for the study of aquatic microbiomes are denaturing gradient gel electrophoresis (DGGE) (Shi et al., 2011; Li et al., 2012), terminal restriction fragment length polymorphism (T-RFLP) (Chen et al., 2010; Li et al., 2011), Sanger sequencing of clone libraries (Chen et al., 2010; Li et al., 2012; Cai and Kong, 2013), and more recently high-throughput sequencing (Fortin et al., 2015; Zwirglmaier et al., 2015; Kurilkina et al., 2016; Lee et al., 2016; Berry et al., 2017).

So far only a few studies have combined high-throughput sequencing-based assessment of cyanobacterial community composition with toxic genotype quantification by qPCR (Fortin et al., 2015; Lee et al., 2016), and even fewer included microcystin measurements (Fortin et al., 2015). These research efforts focused on water bodies in North America (Fortin et al., 2015; Lee et al., 2016; Berry et al., 2017), and Norwegian lakes (Parulekar et al., 2017).

This study aimed to explore possible correlations between microcystin occurrence and factors such as environmental parameters, cyanobacterial community composition, or genetic markers. Specifically, we hypothesized a positive correlation between microcystin synthesis gene copy number and microcystin concentration. Furthermore, we aimed to identify the main microcystin producer in Lake Klostersee and Lake Bergknappweiher.

Here we present the first assessment of the bacteria and cyanobacteria community in two South German lakes during toxic cyanobacterial blooms with Illumina sequencing of the 16S-rRNA gene. Gene quantification by qPCR, phylogenetic identification of the main microcystin producer through sequencing of clone libraries of one microcystin synthesis gene region, and two different microcystin measurement methods underpin the information about community composition.

## Materials and Methods

### Sampling

#### Sampling Area

**FIGURE 1:**
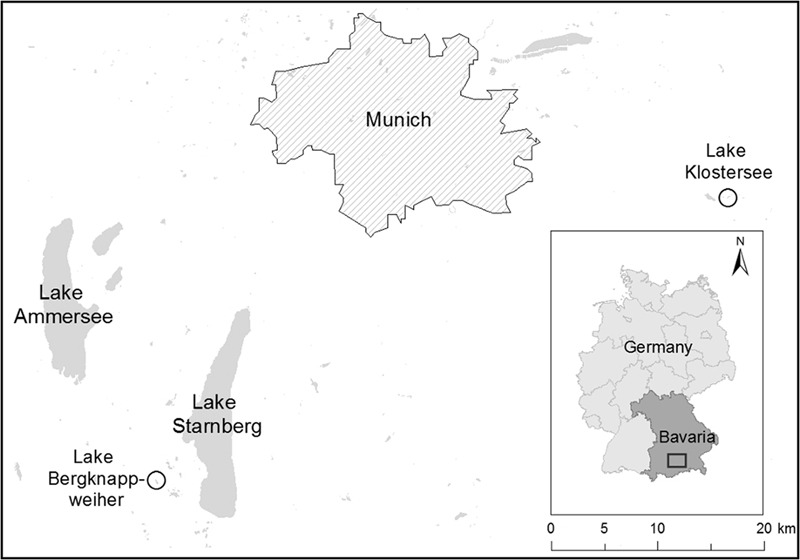
Geographic location of Lake Klostersee and Lake Bergknappweiher.

Both lakes used in this study are known for recurrent cyanobacterial blooms and used for recreational purposes (**Figure 1**). Lake Klostersee (GPS coordinates: 48.08, 11.96) is an artificial eutrophic lake with a maximum water depth of 2.5 m (Teubner et al., 2004; Gesundheitsamt Landratsamt Ebersberg, 2014). It is located 545 m above sea level in Ebersberg, Germany, ca 20 km south–east of Munich. Lake Klostersee was sampled in a biweekly rhythm from May to October 2015. Lake Bergknappweiher (GPS coordinates: 47.85, 11.23) is a small eutrophic and dystrophic lake with a maximum water depth of 2.5 m (Teubner et al., 2004). This lake is located 617 m above sea level about 30 km south-west of Munich, Germany. Lake Bergknappweiher was sampled in a weekly rhythm from August to October 2015.

#### Sampling for DNA, Water Chemistry, and Microcystin Analysis

Surface water samples (1 L) were taken from one near shore sampling point at Lake Klostersee and Lake Bergknappweiher using a 2 L beaker on a pole. From the water sample, 0.5 L was dedicated for DNA analysis, 100 mL was dedicated for microcystin measurements, and 400 mL was dedicated for water chemistry measurements. Water samples were chilled on ice and transported to the laboratory within 2 h, where they were processed immediately (DNA, water chemistry) or frozen at -20°C until sample preparation and extraction or measurement commenced.

### Water Chemistry

Ammonium was measured using an ion chromatograph (Dionex ICS-1100, Thermo Scientific, Waltham, MA, United States) and analyzed using the Chromeleon Software Version 7.2.1.5833 (Thermo Scientific, Waltham, MA, United States). Phosphorus and NO_3_-nitrogen were measured spectrophotometrically (model 150-20; HITACHI, Chiyoda, Japan). Total phosphorus (TP) was measured according to the German standard methods for the examination of water, wastewater, and sludge (DEV, 2013). NO_3_-nitrogen was measured according to Navone (1964). The quotient of inorganic nitrogen and phosphorus (N_inorg._/P) was calculated from measured ammonium, NO_3_-nitrogen, and TP.

### Water Physics

The following physical water parameters were determined using a multi-parameter probe Multi 350i (WTW, Weilheim, Germany): pH, temperature, oxygen concentration, and conductivity. Secchi depth was measured to the nearest 5 cm using a Secchi disk.

### Microcystin Measurements with ELISA

#### Sample Preparation and Extraction

Sample preparation was performed according to the manufacturer’s instructions of a commercially available enzyme-linked immunosorbent assay (ELISA) kit (Beacon Analytical Systems, Portland, OR, United States). Duplicate water samples (5 mL each) were subjected to three freeze-thaw cycles to lyse cells and release microcystins into solution (Zaffiro et al., 2016). This was followed by filtration of the 5 mL water sample using an Acrodisc LC 25 mm syringe filter, 0.45 μm PVDF (Sigma-Aldrich, St. Louis, MO, United States). The filtrate was stored at 4–8°C for a maximum of 12 h.

#### Measurement

Total microcystin (particulate and dissolved microcystin measured together) was measured immunologically in duplicates using a commercially available ELISA kit (Beacon Analytical Systems, Portland, OR, United States). Samples (50 μL) were subjected to the microcystin ELISA kit according to manufacturer’s instructions. The limit of detection (LOD) was 1 μg/L and an upper limit of quantification of 20 μg/L.

### Microcystin Measurements with HPLC

#### Sample Preparation and Extraction

Water samples (90 mL) were subjected to three freeze-thaw cycles, and after centrifugation at 20,000 *g* for 10 min samples were filtered through a 47 mm diameter glass fiber filter with 1.2 μm pore size (Whatman, Maidstone, United Kingdom). Microcystins and nodularin in the filtrate were concentrated by solid phase extraction using Oasis MAX 6 cc cartridges (150 mg, 60 μm particle size, Waters Corporation, Milfort, MA, United States). The filtrate from water samples was applied after conditioning the cartridge with 4 mL methanol and 6 mL water. The cartridge was washed with a 4 mL mixture of methanol-water (5:1, v/v) and microcystin was eluted from the cartridge with 4 mL methanol containing 2% formic acid. The resulting eluent was evaporated by a gentle stream of nitrogen at 40°C and the residue was re-dissolved in 500 μL methanol-water (5:1, v/v). Immediately before chromatographic analysis, the solution was membrane filtered using Acrodisc LC 25 mm syringe filters, 0.45 μm PVDF (Sigma–Aldrich, St. Louis, MO, United States).

#### Measurement

Individual total microcystins and nodularin (particulate and dissolved microcystins and nodularin measured together) were measured together by high-performance liquid chromatography equipped with photodiode array detector (HPLC-PDA). According to Spoof et al. (2010), a sub-3 μm particle-based reversed-phase column was used for chromatography and the mobile phase gradient was optimized for separation of twelve microcystins and nodularin-R (Supplementary Figure S1). HPLC was performed with a Summit^®^ HPLC system equipped with a photo diode array detector (Dionex, Germany). Individual microcystins and nodularin were separated on a 3.0 mm i.d. × 100 mm Kinetex^®^ C18 column (100 Å pore size, 2.6 mm particle size) equipped with a 4 mm i.d. C18 securityguard^TM^ cartridge (Phenomenex, Torrance, CA, United States). The column temperature was set at 40°C. Solvent A consisted of 0.5 mL trifluoroacetic acid and 250 mL methanol adjusted to a final volume of 1000 mL with water, and solvent B was acetonitrile with 0.05% trifluoroacetic acid. The gradient elution program started with 20% B and increased to 40% B in 13 min, then to 63% B in 5 min and subsequently to 100% B in 3 min at a flow rate of 0.5 mL/min. The injection volume was 20 μL and microcystins (MC) were detected at 238 nm. [D-Asp3]-MC-RR, MC-RR, MC-YR, MC-HtyR, MC-LR, [D-Asp3]-MC-LR, MC-WR, MC-HilR, MC-LA, MC-LY, MC-LW, MC-LF and nodularin-R were obtained from Enzo Life Sciences (Germany) and solutions in methanol-water (5:1, v/v) (0.5–2.5 μg/mL) were used for identification and quantification. The recovery was determined by spiking tap water with microcystins (2.5 μg/L). Recoveries were between 64 ± 3% for MC-WR and 123 ± 4% for nodularin-R (mean ± standard deviation, *n* = 5). The LOD for each microcystin variant was 1 μg/L.

### DNA Isolation

A volume of 0.25–0.5 L of lake water was filtered through a 0.2 μm cellulose nitrate filter (Sartorius, Göttingen, Germany) to concentrate cellular organisms on the filter. Filters were stored at -20°C prior to DNA isolation. DNA isolation was performed using a phenol-chloroform based method, which was modified from Fuller et al. (2003). Briefly, 2 mL of lysis buffer (0.75 M sucrose, 0.4 M NaCl, 50 mM Tris, 20 mM EDTA, pH 9.0) and 20 μL lysozyme (50 mg/mL) were added to the filter and incubated while rotating for 30 min at 37°C, followed by heating to 55°C for 10 min. After addition of 2 mL phenol/chloroform/isoamylalcohol (25:24:1 v/v/v) samples were spun at 3,310 g for 5 min at 4°C. Subsequently, the upper phase was separated and mixed with 2 mL of chloroform and spun at 4,000 *g* for 5 min at 4°C. DNA was then precipitated with two volumes of ethanol and 0.1 volume of sodium-acetate 3 M, pH 5.2 for a minimum of 1 h at -20°C. The samples were centrifuged for 45 min at 4,000 *g* at 4°C, and the resulting pellet was washed with 0.5 mL 80% ethanol. DNA was re-suspended in 50–100 μL Tris-EDTA buffer (10 mM Tris, 1 mM EDTA, pH 8.0) and quantified with a NanoVue Plus spectrophotometer (GE Healthcare, Little Chalfont, United Kingdom). The 260 nm/280 nm ratios of DNA samples ranged from 1.68 to 2.11. Isolated DNA was aliquoted and stored at -20°C until further analysis.

### Quantitative Polymerase Chain Reaction

To quantify the *mcyB* gene copies of the genus *Microcystis* and the *mcyE* gene copies of the genus *Dolichospermum*, qPCR was performed. *Planktothrix mcy* genes were not quantified because of the absence of this genus from our Illumina Miseq sequencing data set.

#### *Microcystis mcyB* Quantification

For targeting the *mcyB* gene of *Microcystis* spp., the primers 30F and 108R and a 5′-FAM labeled hydrolysis probe was used (**Table 1**) (Kurmayer and Kutzenberger, 2003). Cycling conditions for this reaction were as follows: 10 min, 95°C; 45 cycles of 15 s, 95°C and 1 min 60°C. A seven-point standard curve was created using five-fold serial dilutions of genomic DNA from *Microcystis aeruginosa* SAG14.85. Calculations were made assuming a genome size for *M. aeruginosa* of 5,842,795 bp as published for *M. aeruginosa* NIES-843 (NC_010296.1) and a single *mcyB* copy per genome. The qPCR amplification and analysis were performed with a magnetic induction cycler and the micPCRv2.4.0 software (Bio Molecular Systems, Sydney, NSW, Australia). We used 25-μL reactions with 0.9 μM primers, 0.25 μM hydrolysis probe, and Taqman Master Mix (Bioron, Ludwigshafen, Germany). All standard curve dilutions and samples were analyzed in triplicates. All environmental DNA samples were diluted one in 20 in Tris-EDTA buffer (10 mM Tris, 1 mM EDTA, pH 8.0). Raw data from qPCR runs had to fulfill the following criteria before gene copy quantification commenced: Samples had to be in the dynamic range of the standard curve. The C_q_ values of triplicates had to be no more than 0.5 cycles apart.

**Table 1:** Information about primers and probes used in this study.

Target gene	Primer name	Sequence 5′ to 3′	Use	Approximately product size (bp)	Source
*mcyE (Microcystis, Dolichospermum*, and *Planktothrix)*	*mcy*E-F2	GAAATTTGTGTAGAAGGTGC	clone library	809	Rantala et al., 2006
	*mcy*E-R4	AATTCTAAAGCCCAAAGACG			
*mcyB (Microcystis)*	30F	CCTACCGAGCGCTTGGG	qPCR	102	Kurmayer and Kutzenberger, 2003
	108R	GAAAATCCCCTAAAGATTCCTGAGT			
	*mcyB* probe	CACCAAAGAAACACCCGAATC TGAGAGG			
*mcyE (Dolichospermum)*	611F	CTAGAGTAGTCACTCACGTC	qPCR	148	Sipari et al., 2010
	737R	GGTTCTTGATAGTTAGATTGAGC			
16S-rRNA	Bakt_341F	**TCGTCGGCAGCGTCAGAT GTGTATAAGAGACAG** CCTACGGGNGGCWGCAG	Illumina amplicon PCR	498	Modified from Herlemann et al. (2011)
	Bakt_805R	**GTCTCGTGGGCTCGGAG ATGTGTATAAGAGACAG** GACTACHVGGGTATCTAATCC			

*Overhanging adapter of primers are shown in bold.*

#### *Dolichospermum mcyE* Quantification

For targeting the *mcyE* gene of *Dolichospermum* spp., the primers 611F and 737R were used (**Table 1**) (Sipari et al., 2010). Cycling conditions for this reaction were as follows: 3 min, 98°C; 15 s, 95°C; 50 s, 64.7°C; 40 cycles, followed by a melting curve analysis of 95°C for 10 min and subsequently a ramp from 65°C to 95°C in 0.5°C increments. A six-point standard curve was created using five-fold serial dilutions of genomic DNA from *Dolichospermum lemmermannii* NIVA-CYA270/1. Calculations were made assuming a genome size of 5,305,670 bp as published for *Dolichospermum sp.* 90 (NC_019427.1), and a single *mcyE* copy per genome. The qPCR amplification and analysis were performed with a BioRad CFX96 cycler and the BioRad CFX Manager software (BioRad, Hercules, CA, United States). We used 20-μL reactions with 0.2 μM primers and SsoAdvanced^TM^ Universal SYBR^®^ Green Supermix (BioRad, Hercules, CA, United States). The qPCR assay was optimized for annealing and extension temperature and time, initial denaturation temperature and time, and primer concentration. All standard curve dilutions and samples were analyzed in triplicates. All environmental DNA samples were diluted one in 20 in Tris-EDTA buffer (10 mM Tris, 1 mM EDTA, pH 8.0). Raw data from qPCR runs had to fulfill the following criteria before gene copy quantification commenced: Samples had to be in the dynamic range of the standard curve. The C_q_ values of triplicates had to be no more than 0.5 cycles apart. Melting curve peaks of samples had to show a single peak at the same temperature and of the same shape as the melting curve peaks of the standard curve dilutions.

### Clone Library and Sanger Sequencing

A clone library of the environmental ca. 809 bp *mcyE* gene fragment was prepared as follows. The initial PCR reaction was performed with the primer pair *mcy*E-F2/*mcy*E-R4 described in Rantala et al. (2006) (see **Table 1**). A 50-μL reaction volume was used with 2.5 U DreamTaq DNA polymerase and DreamTaq buffer (10x) (Fisher Scientific, Schwerte, Germany), 0.75 mM MgCl_2_, 0.2 μM of each primer, 0.15 mM dNTP (Fisher Scientific, Schwerte, Germany), and 0.1 mg/mL BSA (Fisher Scientific, Schwerte, Germany). PCR was performed using a BioRad T100 thermal cycler and the following program: 5 min, 95°C; 15 s, 95°C; 30 s, 56°C; 50 s, 72°C; 35 cycles. The presence and correct size of PCR products were confirmed on a 1.8% agarose gel. The clone library was prepared using the TOPO TA Cloning Kit (Invitrogen, Carlsbad, CA, United States) according to the manufacturer’s instructions. Sanger sequencing services were provided by LGC Genomics (Berlin, Germany). Sequences were quality checked, trimmed, and aligned. Individual and consensus sequences were added to a neighbor-joining tree of published *mcyE* sequences using Geneious version 7.1.5 (Kearse et al., 2012). Sequence data were submitted to GenBank (accessions numbers: MF947220 - MF947378).

### Illumina Sequencing

Material for Illumina sequencing was obtained as described above in the sections on sampling and DNA isolation. An Illumina MiSeq library was prepared according to the Illumina 16S Metagenomic Sequencing Library Preparation guide (Part # 15044223 Rev. B). In short, a metagenomics-sequencing library was prepared using universal bacterial primers with an overhanging adapter targeting the V3 and V4 region of the bacterial 16S-rRNA gene (see **Table 1**). The cycling conditions of the amplicon PCR were: 3 min, 95°C; 30 s, 95°C; 30 s, 55°C; 30 s, 72°C; 25 cycles; 5 min, 72°C. PCR products were purified with Agencourt^®^ AMPure^®^ CP (Beckman Coulter, Krefeld, Germany) magnetic beads according to the manufacturer’s instructions. Index PCR was performed as per the dual indexing principle in the Nextera XT Index Kit (Illumina, San Diego, CA, United States) and the following PCR conditions: 3 min, 95°C; 30 s, 95°C; 30 s, 55°C; 30 s, 72°C; 8 cycles; 5 min, 72°C. For both PCR reactions, the proof reading polymerase Accuzyme Taq (Bioline, Cambridge, United Kingdom) was used in a 50-μL reaction volume, and the presence and correct size of PCR products was confirmed on a 1.8% agarose gel. Index PCR products were purified with Agencourt^®^ AMPure^®^ CP (Beckman Coulter, Krefeld, Germany) magnetic beads according to the manufacturer’s instructions. Samples were quantified using Qubit (Thermo Fisher Scientific, Waltham, MA, United States), pooled, and normalized to a 4 nM concentration. Sequencing was performed bi-directionally using the Illumina MiSeq v3 2x300 paired-end sequencing. Sequence analysis was performed with Usearch version 7 (Edgar, 2010). Sequences were trimmed to 400 bp length and low-quality reads were removed by setting the truncqual parameter to 3. De-replication of files and the removal of singletons were carried out. Operational taxonomic unit (OTU) clustering was carried out at the level of 97% similarity and chimeras were removed afterward. OTU numbering was done with python. OTUs were classified using greengenes (DeSantis et al., 2006) via the SINA aligner and classification tool^1^ (Pruesse et al., 2007). The sequence data was not normalized to avoid loss of information (McMurdie and Holmes, 2014). For rarefaction curves and sequence numbers see Supplementary Information (Supplementary Figure S2 and Supplementary Table S1). The sequence data were submitted to the European Nucleotide Archive (PRJEB21009). The universal bacteria primers used in this study also amplified the chloroplast 16S-rRNA gene sequence of phototrophic eukaryotes due to the cyanobacterial origin of chloroplasts (Sagan, 1967; Schwartz and Dayhoff, 1978). The number of eukaryotic chloroplast reads could not be directly linked to eukaryotic algal relative abundance because eukaryotic algae have a varying number of chloroplasts and chloroplasts have a varying number of genome copies. We identified chloroplasts down to the lowest taxonomic level possible, which was at the taxonomic order level. A classification down to lower taxonomic level was only possible in a few cases (data not shown).

### Statistical Analysis

To assess coverage in the amplicon datasets, rarefaction curves were plotted. Pearson’s correlation coefficient was utilized to shed light on correlations between *mcyB* gene abundance, microcystin concentrations, environmental factors, bacterial communities, and cyanobacterial taxa. All statistical analyses were done with Past v3.06 (Hammer et al., 2001).

## Results

### Microbial Community Composition

#### Total Bacterial Community Composition (BCC) at the Phylum Level

**FIGURE 2:**
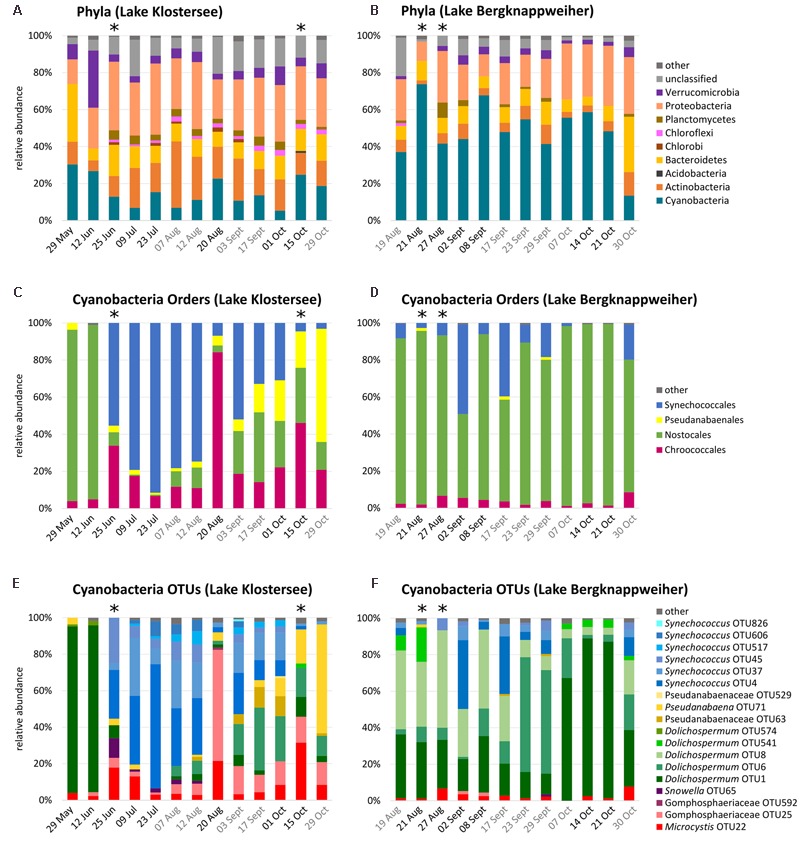
Bacterioplankton community composition in Lake Klostersee (left) and Lake Bergknappweiher (right) (relative abundances < 1% shown as “other”). Dates in black indicate VSBs. Dates in grey indicate the absence of VSBs. Asterisks indicate < 1,000 sequences. Note different intervals on *x*-axis. **(A,B)** Percent relative abundances of bacterial taxa at the phylum level. **(C,D)** Percent relative abundances of cyanobacterial taxa at the order level. **(E,F)** Percent relative abundances of cyanobacterial taxa at the level of OTUs (97% sequence identity).

The overall composition of bacterial phyla in Lake Klostersee was rather stable over the sampling period. The most abundant phyla throughout the sampling period were Proteobacteria, Actinobacteria, and Cyanobacteria (**Figure 2A**). The sample from 12 Jun 2015 with high abundance of Verrucomicrobia and the sample from 29 May 2015 with high abundances of Bacteroidetes formed exceptions.

Like in Lake Klostersee, in Lake Bergknappweiher the overall composition of bacterial phyla was relatively stable over the sampling period (**Figure 2B**). Exceptions were the samples from 21 Aug and 30 Oct 2015 with high and low relative abundances of Cyanobacteria, respectively. The single most abundant phylum in Lake Bergknappweiher throughout the sampling period was Cyanobacteria with an exception at the end of the sampling period on 30 October 2015 when Bacteroidetes and Proteobacteria were more abundant than Cyanobacteria. The second most abundant phylum in this lake was Proteobacteria.

#### Cyanobacterial Community Composition

The community composition of Cyanobacteria in Lake Klostersee changed considerably over the sampling period (**Figures 2C,E**). We observed a major shift from Nostocales, which were dominated by a single *Dolichospermum* OTU, at the beginning of the sampling period to a dominance of Synechococcales, which were represented by up to six *Synechococcus* OTUs, from late June to mid-August. A sudden peak of Chroococcales followed this on 20 August 2015. After that, we observed a more balanced distribution of the main cyanobacterial orders with ever decreasing relative abundances of Synechococcales and increasing relative abundances of Pseudanabaenales.

In Lake Bergknappweiher, Cyanobacteria made up the largest fraction of the total bacterial community. Mean relative abundance of Cyanobacteria in Lake Bergknappweiher was more than three times higher than in Lake Klostersee. The community composition of Cyanobacteria in Lake Bergknappweiher changed during the sampling period, in particular at the OTU level (**Figures 2D,F**). Nostocales, which were represented by up to five *Dolichospermum* OTUs, were very dominant throughout the entire sampling period. In mid-October, a single *Dolichospermum* OTU dominated the Nostocales population. Peaks in omnipresent Synechococcales were evident beginning- and mid-September. *Microcystis* was present in every sample.

#### Community Composition of Main Heterotrophic Phyla

**FIGURE 3:**
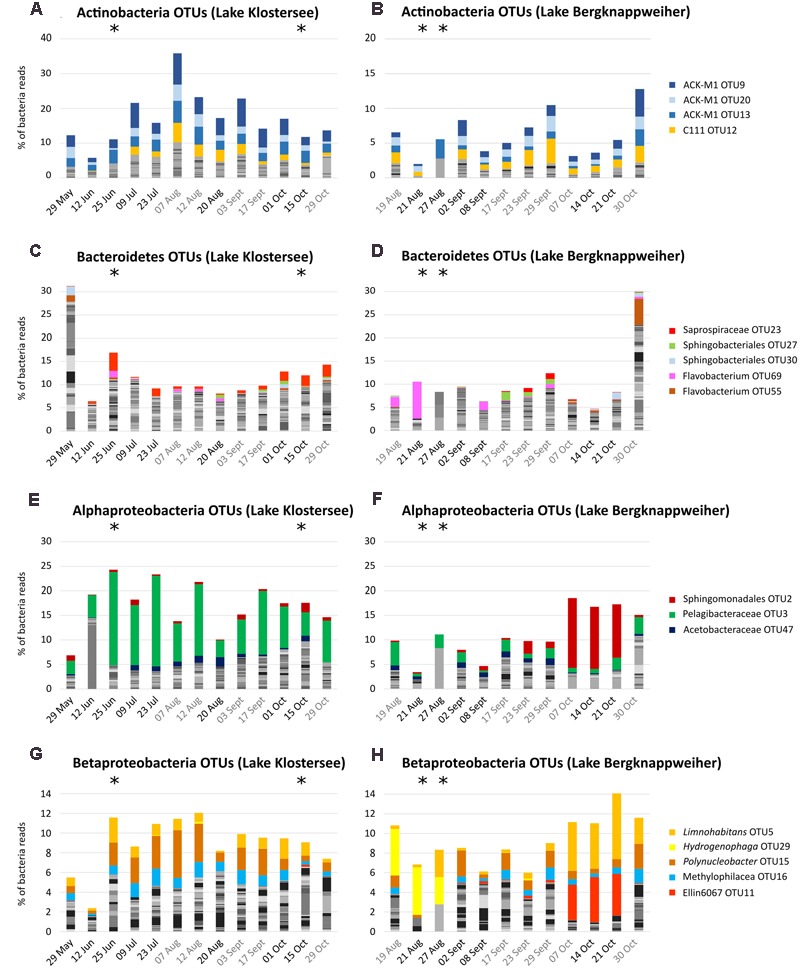
Non-cyanobacteria community composition in Lake Klostersee (left) and Lake Bergknappweiher (right) at the OTU level (97% sequence identity). Dates in black indicate VSBs. Dates in gray indicate the absence of VSBs. Note different intervals on *x*-axis. **(A,B)** Actinobacteria **(C,D)** Bacteroidetes. **(E,F)** Alphaproteobacteria **(G,H)** Betaproteobacteria (note different *y*-axis).

Operational taxonomic unit level analysis revealed that Actinobacteria in Lake Klostersee and Lake Bergknappweiher displayed a similar community composition, which remained stable over the sampling period (**Figures 3A,B**). Three members of the order Actinomycetales (OTU9, 20, and 13) and one Acidimicrobiales (OTU12) dominated the Actinobacteria community. All four OTUs showed a significant negative correlation with the cyanobacterial genus *Dolichospermum* and a positive correlation with the genus *Synechococcus* (Supplementary Table S2).

In contrast to this, the many members of the highly diverse phylum Bacteroidetes showed a highly dynamic community composition (**Figures 3C,D**). OTU23, a member of the family Saprospiraceae correlated positively with *Microcystis* and negatively with the genus *Dolichospermum* (Supplementary Table S2).

Proteobacteria showed distinctively different patterns in the two lakes. In Lake Klostersee, Alpha- and Betaproteobacteria formed communities, which were stable over the sampling period (**Figures 3E,G**). By contrast, in Lake Bergknappweiher those classes showed considerable temporal variability in their community composition (**Figures 3F,H**). The dominant OTU in the Alphaproteobacteria community was a member of the order Sphingomonadales, OTU2, and a member of the family Pelagibacteraceae, OTU3. The first correlated positively with *Dolichospermum* (especially OTU1 in Lake Bergknappweiher) and the latter correlated positively with *Synechococcus* and negatively with *Dolichospermum* (Table S2). The dominant Betaproteobacteria were three different OTUs from the family Comamonadaceae (the genus *Limnohabitans, Hydrogenophaga*, and *Polynucleobacter*) and a member of the family Methylophilaceae and the order Ellin60607 (almost absent in Lake Klostersee). *Limnohabitans* OTU5 and Ellin60607 OTU11 correlated positively with *Dolichospermum* OTU1 (Supplementary Table S2). None of the dominant non-cyanobacterial OTUs in either phylum correlated positively with microcystin concentration.

#### Chloroplast Community Composition

**FIGURE 4:**
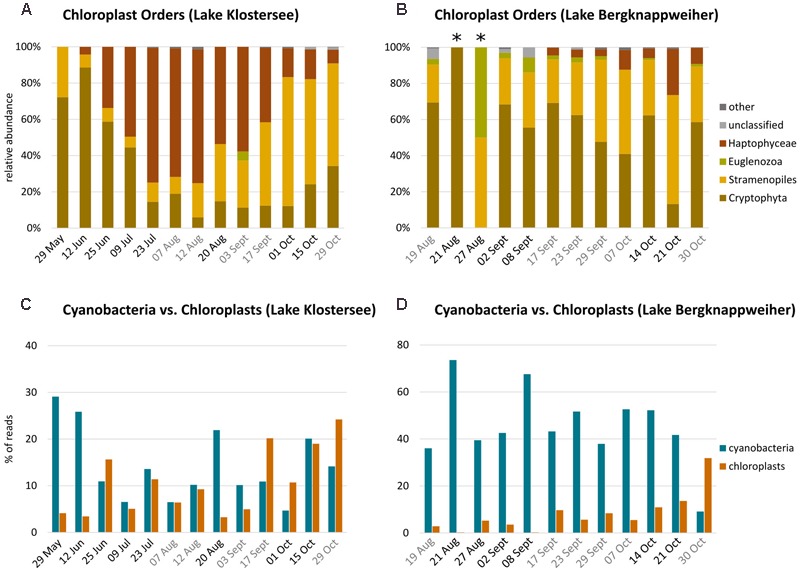
Chloroplast community composition and percent sequence reads in Lake Klostersee (left) and Lake Bergknappweiher (right). Note different intervals on *x*-axis. **(A,B)** Relative abundance of chloroplast sequences at the order level (relative abundances < 1% shown as “other”, asterisks mark samples with low sequence number). Dates in black indicate VSBs. Dates in gray indicate the absence of VSBs. **(C,D)** Relative abundance of Cyanobacteria vs. chloroplast percent sequence reads (note different *y*-axis).

In the samples from Lake Klostersee, chloroplasts from Cryptophyta, Stramenopiles, and Haptophyceae dominated. Noteworthy abundances of Euglenozoa occurred only on 03 September 2015 (**Figure 4A**). The percentage of reads identified as being from chloroplasts showed a weak negative correlation with the relative abundance of Cyanobacteria in Lake Klostersee (*r* = -0.20) (**Figure 4C**). In samples from Lake Bergknappweiher, chloroplasts from Cryptophyta and Stramenopiles dominated most of the time with some Euglenozoa and Haptophyceae (**Figure 4B**). In Lake Bergknappweiher the percentage of reads identified as chloroplast showed a strong negative correlation with the relative abundances of Cyanobacteria (*r* = -0.80) (**Figure 4D**).

In total, the relative abundance of Cyanobacteria and percentage of chloroplast reads were negatively correlated (Supplementary Table S3). The different chloroplast orders showed distinctive correlations with environmental parameters and cyanobacterial orders (Supplementary Table S3). None of the chloroplast orders showed any correlation with microcystin concentration or *mcyB* abundance.

#### Comparison of Lake Bergknappweiher and Lake Klostersee BCC

**FIGURE 5:**
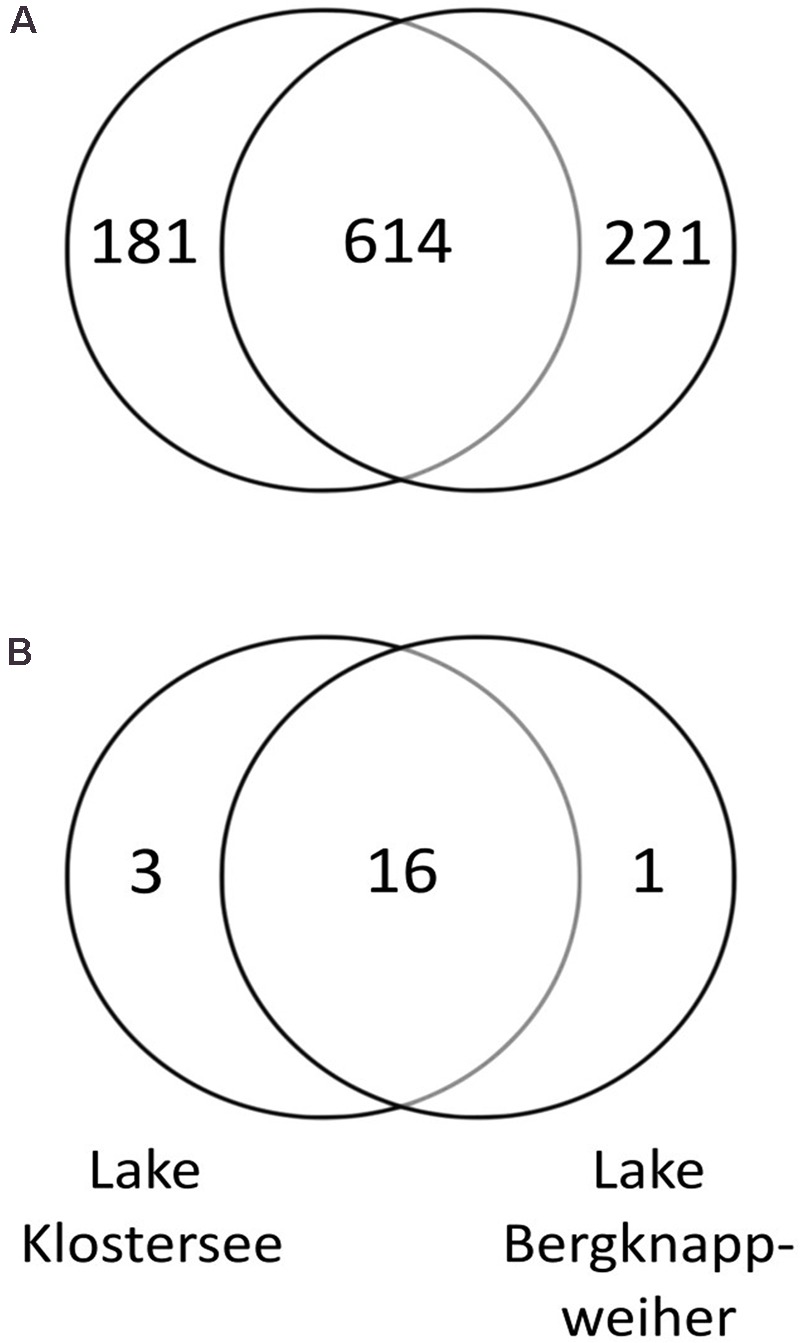
**(A)** Number of unique and shared bacterial OTUs between Lake Bergknappweiher and Lake Klostersee. **(B)** Number of unique and shared cyanobacterial OTUs between Lake Bergknappweiher and Lake Klostersee. Chloroplasts sequences were excluded from both analyses. Circles not drawn to proportion.

A total of 1,076 different OTUs were identified in this study. Of those OTUs 60 OTUs were chloroplast sequences and excluded from the bacterioplankton analysis. We identified 795 different bacterial OTUs in Lake Klostersee out of which approximately one quarter (22.77 %) were unique to this lake and not present in Lake Bergknappweiher. In Lake Bergknappweiher, we identified 835 different bacterial OTUs out of which approximately one quarter (26.47%) were unique to this lake. The OTUs unique to the lakes were rare and together made up only a small fraction of the microbiome in terms of relative abundance (4.43% in Lake Klostersee and 2.73% in Lake Bergknappweiher). A common set of 614 OTUs was present in both lakes (see **Figure 5A**). This means that the lakes shared approximately three quarters of bacterial taxa (73.53% of Lake Bergknappweiher and 77.23% of Lake Klostersee). Those common OTUs contributed to over 95% of the microbiome in terms of relative abundance.

In this study, 20 different OTUs belonged to the phylum Cyanobacteria. In Lake Bergknappweiher, 17 different cyanobacterial OTUs were present out of which only one was unique to this lake. In Lake Klostersee, we found 19 different cyanobacterial OTUs out of which only three were unique to this lake. Hence, a common set of 16 cyanobacterial OTUs was found in both lakes (see **Figure 5B**). Thus, the lakes shared the majority of cyanobacterial OTUs (for Lake Klostersee 84.21% and for Lake Bergknappweiher 94.12%).

One of the most abundant cyanobacterial OTUs was *Dolichospermum* (OTU1). It was the main contributor to visible surface blooms (VSBs) in both the spring sampling points in Lake Klostersee and the autumn sampling points in Lake Bergknappweiher (**Figures 2E,F**). During those times, it accounted for over 85% of the whole cyanobacterial population. A BLAST search revealed *Dolichospermum flos-aquae* (accession number KY327796.1, identity 100%) as the closest match for *Dolichospermum* OTU1. *Dolichospermum flos-aquae* has previously been found to dominate in Lake Bergknappweiher (Teubner et al., 2004).

### Microcystin-Producing Cyanobacteria

#### Toxigenicity

**FIGURE 6:**
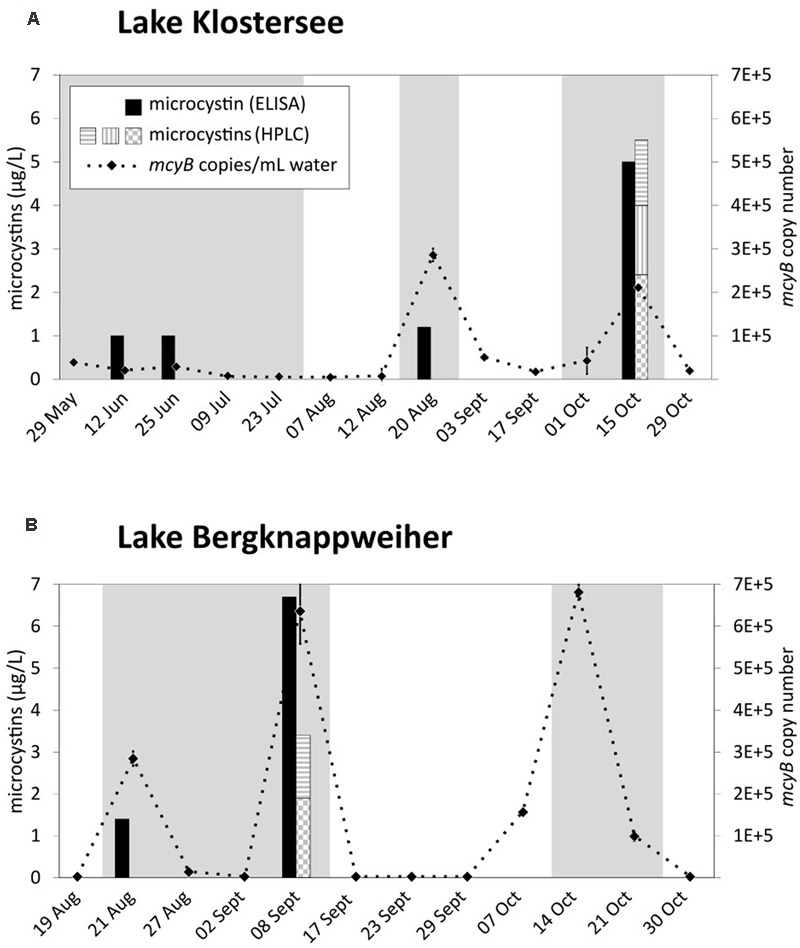
Microcystins and *mcyB* copy number over the bloom period 2015 in **(A)** Lake Klostersee and **(B)** Lake Bergknappweiher. Gray background indicates visible surface blooms. Microcystins (HPLC): Horizontal gray stripes indicate microcystin-LR, vertical gray stripes indicate microcystin-YR, and gray check indicates microcystin-RR. Error bars indicate standard error of the mean. If error bars are not visible, they are smaller than the symbols.

To gauge bloom toxigenicity, the microcystin biosynthesis genes *mcyB* and *mcyE* were assessed. The LOD for *Microcystis mcyB* was 6.12 × 10^2^ copies/mL water. For *mcyB* gene copy numbers see **Figure 6** and Supplementary Table S4. The qPCR reaction detecting *mcyE* was specific for *Dolichospermum* (Supplementary Figure S3). The LOD was 3.38 × 10^3^ copies/mL water. All environmental samples gave a negative result for *Dolichospermum mcyE* (Supplementary Table S4). In both lakes, peaks in *mcyB* copy number coincided with VSBs (**Figure 6**).

#### Diversity and Origin of *mcyE* Genes

**FIGURE 7:**
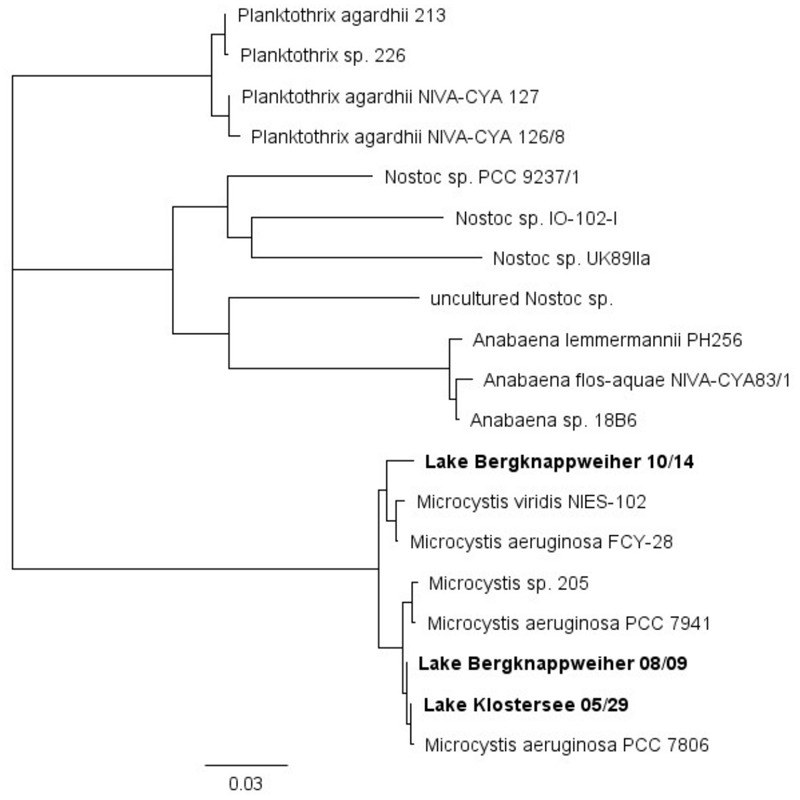
Neighbor-joining tree based on published partial and full *mcyE* sequences and *mcyE* consensus sequences obtained from clone libraries in this study (in bold).

We assessed the diversity and taxonomic origin of the microcystin biosynthesis gene *mcyE* for one sampling point in Lake Klostersee and two sampling points in Lake Bergknappweiher. In the Lake Klostersee sample from 29 May 2015 we identified 82 highly similar partial *mcyE* sequences with a pairwise percent identity of 98.8%. In the Lake Bergknappweiher samples from 08 September and 14 October 2015 a total of 61 and 16 highly similar partial *mcyE* sequences were identified with a pairwise percent identity of 98.3 and 98.5%, respectively. All of the partial *mcyE* sequences and their consensus sequences clustered with *mcyE* sequences from *Microcystis* sp. in a neighbor-joining tree with published *mcyE* sequences (**Figure 7**). None of the sequences clustered with *mcyE* sequences from other genera such as *Dolichospermum* or *Planktothrix*, indicating that all *mcyE* sequences detected originated from *Microcystis* sp.

### Physico-Chemical Parameters

For microcystin concentrations in both lakes see **Figure 6** and Supplementary Table S5. For further physico-chemical water parameters see Supplementary Table S5.

In both lakes microcystin measurements by ELISA and HPLC correlated strongly [Pearson’s *r* (*r*) = 0.89] and showed a positive correlation with *mcyB* copy number (Supplementary Table S6). All three parameters were positively correlated with TP and negatively with N_inorg._/P (Supplementary Table S6). The correlations of microcystins with NO_3_-nitrogen, however, were different for both lakes. In Lake Klostersee, microcystin concentrations were negatively correlated with NO_3_-nitrogen (ELISA *r* = -0.47, HPLC *r* = -0.42) while in Lake Bergknappweiher they were positively correlated with NO_3_-nitrogen (ELISA *r* = 0.51, HPLC *r* = 0.41). In both lakes, actual microcystin measurements coincided with VSBs. However, not all VSBs were characterized by detectable microcystin concentrations (**Figure 6** and Supplementary Table S5).

Correlation analysis revealed a clear two-way division between cyanobacterial genera. The relative abundance of OTUs belonging to the genus *Dolichospermum* showed a very strong negative correlation with NO_3_-nitrogen and a negative correlation with TP (Supplementary Table S7). All other genera correlated positively with NO_3_-nitrogen and showed positive or no correlation with TP (Supplementary Table S7). *Microcystis* showed a positive correlation with TP and NO_3_-nitrogen (Supplementary Table S7).

## Discussion

In this study, we identified environmental parameters that correlated with microcystin occurrence. Of the environmental parameters recorded in this study, N_inorg_/P and TP showed a correlation with microcystin occurrence. In line with our hypothesis, we showed that *mcyB* gene copy number correlated positively with microcystin concentration. We identified *Microcystis* sp. as the sole microcystins producer. *Microcystis* sp. was not very abundant and never dominated the cyanobacterial community. Furthermore, we showed that the BCC was relatively stable at the phylum level throughout the sampling period, but temporal changes in the community composition at the OTU level were evident. Our study adds to the few previous studies that combine various community aspects with toxicity (Fortin et al., 2015; Berry et al., 2017; Parulekar et al., 2017) and differs from previous work where either *Microcystis* was dominating the Cyanobacteria community (Fortin et al., 2015) or only the phylum Cyanobacteria was resolved down to OTU level (Fortin et al., 2015; Parulekar et al., 2017).

### Microbial Community Composition

#### Phylum Level vs. OTU Level Distribution

The appearance of a VSB did not coincide with massive changes in BCC at the phylum level, which was mostly stable throughout the sampling period. This partly contradicts other studies, which have found a low resistance but high resilience of the BCC toward cyanobacterial blooms (Li et al., 2012) or temporal changes in phylum level distribution (Parulekar et al., 2017). However, we observed considerable temporal changes in bacterial community composition at the level of order and even more at the OTU taxonomic level (**Figures 2, 3**). These data show that it is important to evaluate the BCC at the OTU level in addition to the phylum or order level because most of the temporal variation in bacterial community composition occurred at the OTU level.

#### Community Composition of Main Heterotrophic Phyla

Interestingly, major bacterial phyla exhibited not only highly different levels of diversity but also different degrees of temporal variation in community composition (see **Figure 3**). In our study, the community of Actinobacteria was stable throughout the sampling period and similar between the lakes. In contrast to our findings, Berry et al. (2017) described an Actinobacteria community that was highly dynamic over time and showed a strong correlation to bloom dynamics. This link to cyanobacteria dynamics might also explain our contrasting observation of a relatively stable Actinobacteria community. The changes in total cyanobacteria relative abundance over time observed in our study were much smaller than the ones observed by Berry et al. (2017). In our study, the cyanobacterial community composition was associated with some species-specific correlations with non-cyanobacterial taxa. However, we observed less bloom-induced changes in the bacterial community composition than others did (Woodhouse et al., 2016; Berry et al., 2017). Most notably the frequently reported bloom related changes in Actinobacteria community composition were not observed here. Possible explanations are low bloom intensity or low abundance of the microcystin producer *Microcystis*. The main Actinobacteria OTUs (**Figure 3**) belong to the most common taxa in freshwater. This phylum has been shown to be more successful under lower nutrient conditions (Newton et al., 2011), which is in line with our results that show a two times higher relative abundance of Actinobacteria in the lake with the lower nutrient load (Lake Klostersee) compared to the lake with the higher nutrient load (Lake Bergknappweiher).

The drastic temporal variations in bacterial community composition within the highly diverse phylum Bacteroidetes has previously been described in the context of toxic cyanobacteria blooms (Woodhouse et al., 2016). The link between Bacteroidetes and Cyanobacteria is also evident in our study. The three most abundant OTUs of the phylum Bacteroidetes were members of the order Sphingobacteriales (OTU23, OTU27, and OTU30) (see **Figures 3C,D**). Members of this taxon, such as Saprospiraceae, are known for their ability to degrade toxins and other cyanobacterial secondary metabolites (Mcilroy and Nielsen, 2014; Li et al., 2017). Moreover, certain Sphingobacteriales lyse cyanobacteria by means of algicidal metabolites, and members of the Saprospiraceae are known to even prey on cyanobacteria (Lewin, 1997). Thus, the positive correlation of Saprospiraceae OTU23 with the toxin producer *Microcystis* emphasizes the close interaction between members of the toxin producing cyanobacteria and heterotrophic bacteria (Supplementary Table S2).

The different patterns of Alpha- and Betaproteobacteria in the two lakes studied could be explained by the fact that environmental parameters in Lake Bergknappweiher, such as nutrients and pH fluctuated more than in Lake Klostersee (Supplementary Table S5). The peculiar and constant community composition of both Alpha- and Betaproteobacteria in Lake Bergknappweiher between 07 October and 21 Octoer 2015 (**Figures 3F,H**) coincided with the overwhelming dominance of *Dolichospermum* OTU1 (**Figure 2F**). One of the most abundant Alphaproteobacteria during this time was a member of the order Sphingomonadales (OTU2) (see **Figure 3F**). Several members of this taxon are able to degrade microcystins (Li et al., 2017), and the high relative abundance of OTU2 between 07 October and 21 October 2015 might hint toward the presence of a similar secondary metabolite possibly produced by *Dolichospermum* or the presence of a yet undetectable microcystin isoform. This would explain the peak in *mcyB* gene copy concentration (qPCR data) that lacks a concurrent peak in microcystin concentration (ELISA and HPLC data) during this period (**Figure 6**). One of the dominant Betaproteobacteria belonged to the genus *Limnohabitans*. *Limnohabitans* can utilize algal derived substrates possibly produced by *Dolichospermum* (Šimek et al., 2011). The fact that high relative abundances of Sphingomonadales and *Limnohabitans* were not observed in Lake Klostersee, while the same *Dolichospermum* dominated the cyanobacterial community (29 May and 12 June 2015), can be explained by the overall lower relative abundance of cyanobacteria in Lake Klostersee.

The data from our study complement other studies that have looked at the cyanobacterial bloom associated BCC (Wu et al., 2007; Shi et al., 2011; Lee et al., 2016; Berry et al., 2017). This additional information from aquatic habitats in other geographic locations is important to distinguish patterns in BCC that are influenced by environmental factors (Bacteroidetes and partly Proteobacteria in our study) from those that are influenced by disturbances such as cyanobacterial blooms (Actinobacteria in Berry et al., 2017). This is a prerequisite to understanding the dynamics in bloom associated bacterial community composition and better gauge environmental implications of toxic cyanobacterial blooms. The sampling period in this study spanned a single season in two lakes. Nevertheless, our results show that the high temporal resolution, which was higher than in many studies and which was called for by Parulekar et al. (2017), was necessary for tracing BCC dynamics at the OTU level at least for some phyla.

#### Diversification within the Dominant Cyanobacterial Genera

Analysis at the lower taxonomic levels revealed that the cyanobacterial community composition was highly dynamic throughout the sampling period. Nevertheless, a single genus was able to dominate the cyanobacterial community for several months. In Lake Klostersee this was *Synechococcus* and in Lake Bergknappweiher *Dolichospermum* (**Figures 2E,F**). Analysis of community composition at the OTU level showed that several different OTUs contributed to the high relative abundance of the dominant genus, but the dominance of any single OTU never lasted longer than about 2 weeks. Top–down control by cyanophages, grazers or algicidal bacteria may prevent any one OTU from becoming dominant over an extended period. Any dominant species might be decimated according to the kill-the-winner hypothesis allowing competitors to thrive (Thingstad and Lignell, 1997). This idea is supported by findings presented by Yoshida et al. (2006) who showed that cyanophages are largely strain specific when infecting their host. Thus, a top down control by viruses can stimulate host diversity (Thingstad and Lignell, 1997; Weinbauer and Rassoulzadegan, 2004; Zwart et al., 2005). Correspondingly, selective feeding on even closely related cyanobacteria strains by grazers has been shown (Zwirglmaier et al., 2009), which might prevent one OTU from becoming dominant and further promote diversification within the dominant cyanobacterial genus. In addition, different algicidal bacteria are capable of lysing cyanobacteria in a species-specific manner (Redhead and Wright, 1978; Rashidan and Bird, 2001). Those algicidal bacteria can shape the cyanobacterial community composition and lead to the decline of algal blooms (Rashidan and Bird, 2001). The filamentous cyanobacterium *Dolichospermum* has been shown to be particularly sensitive to lysis by co-occurring algicidal bacteria (Redhead and Wright, 1978; Rashidan and Bird, 2001). Algicidal bacteria thus might have caused the drop in *Dolichospermum* relative abundance in Lake Klostersee after the 12 June 2015 (**Figure 2E**). This conclusion is supported by the fact that Illumnia Miseq sequencing data set contained bacteria that have previously been shown to have algicidal activity, such as members of the order Sphingobacteriales and the family Saprospirace (Lewin, 1997) (**Figure 3**) and the genera *Pseudomonas* sp. and *Microbacterium* sp. (Redhead and Wright, 1978; Chen et al., 2012). The fact that the potential algicidal bacteria Sphingobacteriales and Saprospiracea (OTU23, 27, and 30) are amongst the most abundant Bacteroidetes (**Figures 3C,D**) hints toward a potential role in regulation of the bacterial community composition. In addition, less abundant Alpha- and Gammaproteobacteria from the family Rhodobacteraceae, Aeromonadaceae, and Enterobacteriacea were identified in this study, and members of those families have been associated with algicidal activity against cyanobacteria (Redhead and Wright, 1978; Chen et al., 2012; Yang et al., 2013; Liao et al., 2014).

#### Chloroplasts

The negative correlation of cyanobacteria and chloroplast reads we observed on a temporal scale (**Figures 4C,D**) has been noted before (Zwirglmaier et al., 2015), albeit in a spatial distribution. This contrasting distribution might be due to the occupation of a somewhat similar ecological niche by eukaryotic algae and cyanobacteria and competition for similar resources. We found a negative correlation of chloroplast reads with water temperature (*r* = -0.54). Our results support the findings of others who found that eukaryotic phytoplankton is better adapted to lower temperatures than cyanobacteria (Butterwick et al., 2005; de Senerpont Domis et al., 2007; Jöhnk et al., 2008).

#### Comparison of Lake Bergknappweiher and Lake Klostersee BCC

Both lakes sampled displayed a distinct bacterial community composition at the phylum level (**Figures 2A,B**) albeit sharing a majority of OTUs (**Figure 5**). OTUs unique to each lake contributed less than 5% to the BCC in terms of relative abundance. This means that the difference in relative abundance of taxa, rather than their presence or absence accounted for the dissimilarity in BCC of both lakes, a conclusion similar to the one drawn by Staley et al. (2013). Regardless of their low relative abundances, the taxa unique to a lake might still be important because rare taxa were proposed to play an important role in shaping of future communities (Sogin et al., 2006).

The relative close geographic proximity of the lakes studied here is not sufficient to explain the large common microbiome on the OTU level. Even adjacent and interconnected lakes were found to share only a much smaller fraction of their microbiome, and environmental conditions were suggested as drivers for differential prokaryotic community composition (Zwirglmaier et al., 2015).

The most striking difference in the bacterial community composition between Lake Klostersee and Lake Bergknappweiher is the higher abundance of cyanobacteria in the latter. OTU level analysis revealed that those cyanobacteria are for the most part *Dolichospermum* species. This genus is diazotrophic and was the only cyanobacterial genus, which correlated negatively with NO_3_-nitrogen (Supplementary Table S7). It is thus less prone to suffer from nitrogen limitation and therefore can take advantage of the high TP concentrations in Lake Bergknappweiher compared to Lake Klostersee. This shows that environmental conditions shape differences in lakes’ community composition.

#### Taxonomic Classification

In this study, we used the 16S-rRNA gene for taxonomic classification of bacteria. The 16S-rRNA gene is a suitable marker for identification of prokaryotes at the species level and has been widely used for investigation of cyanobacterial diversity (Kirkwood et al., 2008; Foster et al., 2009; Kormas et al., 2010; Lymperopoulou et al., 2011; Zwirglmaier et al., 2015; Ruber et al., 2016). It should be noted that some bacteria carry more than one copy of the 16S-rRNA gene (Pei et al., 2010). Schirrmeister et al. (2012) found *Nostoc* spp. and *Dolichospermum variabilis* to carry four 16S-RNA gene copies while *M. aeruginosa* NIES-843 only carried two. The number of 16S-rRNA gene copies cannot necessarily be inferred from the genus: for example, different members of the genus *Synechococcus* were found to carry either one or two copies of the 16S-rRNA gene in the same study (Schirrmeister et al., 2012). Nevertheless, Parulekar et al. (2017) showed that *Dolichospermum flos-aquae* was not overestimated by high-throughput sequencing compared to microscopic analysis.

### Microcystin-Producing Cyanobacteria

#### Correlations with Microcystin Occurrence

*Microcystis* was present in almost every sample from Lake Klostersee and Lake Bergknappweiher and its highest relative abundances on 25 Jun, 20 Aug, and 15 October 2015 coincided with microcystin detections (**Figure 6A**). The highest relative abundances of *Microcystis* in Lake Bergknappweiher on 27 August and 30 October 2015 did not coincide with positive microcystin results (**Figure 6B**). A strong positive correlation of relative abundance of *Microcystis* sp. (based on the 16S rRNA gene) with microcystin concentration was found in Lake Klostersee (ELISA *r* = 0.70, HPLC *r* = 0.68) but not in Lake Bergknappweiher. There are several possible explanations for these observations apart from the relative nature of the Illumina sequence data. First, a shift in the ratio of *mcyB* genotypes and *mcyB* gene free genotypes during the sampling period might have occurred. Genotypes containing *mcyB* and *mcyB* gene free members of the same species not only co-occur in natural environments but also vary in their contribution to the total population (Kardinaal et al., 2007; Briand et al., 2008; Sabart et al., 2010; Martins et al., 2011). Second, microcystin biosynthesis genes may not be expressed constitutively, which still awaits independent confirmation (Wood et al., 2011). Regulation of microcystin biosynthesis genes at the transcriptional level has been described in a laboratory study (Sevilla et al., 2008; Pimentel and Giani, 2014; Scherer et al., 2017) and in the environment (Penn et al., 2014). A third explanation is the possible presence of *mcyB* genotypes with microcystin biosynthesis gene clusters that are inactivated by mutations. Such non-functional gene clusters have been described in environmental studies (Christiansen et al., 2006; Christiansen et al., 2008; Ostermaier and Kurmayer, 2009).

For analysis of total microcystin in surface water samples extracellular as well as intracellular microcystins have to be considered. Extraction of intracellular microcystins from the cyanobacteria is thus a critical step for the quantitative analysis of microcystin. Microcystin levels detected in this study were far below the action level of 30 μg/L recommended by the German Federal Environment Agency for recreational water (Bundesgesundheitsblatt, 2015). Four samples exhibited a microcystin level above the LOD of the ELISA (1.0–1.4 μg/L). In these samples, specific microcystins were not detected by HPLC, probably because they were below the LOD of the HPLC method. Higher microcystin levels detected by ELISA in two samples (5.0 and 6.7 μg/L) were confirmed by HPLC (5.5 and 3.4 μg/L). Deviations observed for results obtained by ELISA and HPLC are due to different principles of detection and possibly differential affinities of the ELISA antibodies to various microcystin isoforms. Other potentials and limitations of different methods for microcystin analysis have been reported (Sangolkar et al., 2006; Kaushik and Balasubramanian, 2013; Schmidt et al., 2014; Meriluoto et al., 2017).

#### Low Abundant Microcystin Producer

In this study, we identified the microcystin-producing cyanobacterium without the need for time-consuming isolation and cultivation. *Microcystis* sp. was present in much lower abundances than *Dolichospermum* spp. in both lakes (**Figures 2E,F**). *Planktothrix* spp. were absent in both lakes. We found no evidence that any of the *Dolichospermum* OTUs identified by high-throughput sequencing carried the genes for microcystin biosynthesis. Data from qPCR as well as data from clone libraries suggested that in both lakes *Microcystis* sp. was the only microcystin producer present. Those findings are in line with other studies, which found that the dominant microcystin producer is not necessarily the dominant cyanobacterial species in a water body (Dadheech et al., 2014). Similar to our study, Lee et al. (2015) identified low abundant *Microcystis* sp. as the sole microcystin producer. More awareness about the possibility of low abundant microcystin producers is required to avoid underestimating toxicity risks in blooms that predominantly contain non-toxic species.

### Physico-Chemical Parameters

The positive correlation of TP with microcystin concentration and microcystin synthesis gene copy number observed in this study has previously been reported (Amer et al., 2009; Rinta-Kanto et al., 2009). A negative correlation between the copy number of a microcystin biosynthesis gene and NO_3_-nitrogen like the one we discovered in Lake Klostersee was shown before (Rinta-Kanto et al., 2009). On the other hand, the positive correlation of those factors as observed in Lake Bergknappweiher was also demonstrated in another study (Yoshida et al., 2007). Our results and previous studies, therefore, suggest that the influence of NO_3_-nitrogen may vary in different water bodies depending on the limiting factor in a particular lake.

We found a positive correlation between *mcyB* copy number (based on qPCR results) and microcystin concentration, which is in agreement with previous work (Ostermaier and Kurmayer, 2010; Martins et al., 2011; Al-Tebrineh et al., 2012). Whether the *mcy* copy number quantified by qPCR can be used as a proxy for microcystin occurrence or not, is still under discussion (Pacheco et al., 2016). The results presented in this study do not settle the matter definitively but indicate it might be possible.

## Conclusion

For the first time, high-throughput sequencing was used to reveal differential temporal variations in the microbial community composition during toxic cyanobacterial blooms in two lakes in South Germany. A focused analysis revealed that the microcystin producing species never dominated the bloom. In addition, *mcyB* gene copy number correlated positively with microcystin concentration. These findings are relevant for risk assessment and therefore need to be the subject of further research. The collective results presented in this study will aid in future risk assessment for recreational waters and help to put microcystin measurements into greater ecological perspective.

## Author Contributions

PS designed and performed sampling and experiments, analyzed and interpreted data, and wrote the manuscript. ADM did data acquisition, quality control, and analysis, and critically reviewed the manuscript. AM and RS were involved in data acquisition and quality control of data. AM also wrote parts of the manuscript. UR and JG were involved in designing the study and critically reviewed the manuscript. KZ contributed to the design of the work and structural composition of the manuscript.

## Conflict of Interest Statement

The authors declare that the research was conducted in the absence of any commercial or financial relationships that could be construed as a potential conflict of interest.

## Footnotes

1 www.arb-silva.de
