# Supplementary material for: Temporal Dynamics of the Microbial Community Composition with a Focus on Toxic Cyanobacteria and Toxin Presence during Harmful Algal Blooms in Two South German Lakes
Source: Front Microbiol. 2017 Dec 4;8:2387. doi: 10.3389/fmicb.2017.02387 (PMC5722842; doi:10.3389/fmicb.2017.02387)
Supplement: Supplementary file 1 [file Data_Sheet_1.PDF]

Supplementary Material

Temporal dynamics of the microbial community composition with a focus on toxic cyanobacteria and toxin presence during harmful algal blooms in two South German lakes

Pia I. Scherer, Andrew D. Millard, Andreas Miller, Renate Schoen, Uta Raeder, Juergen Geist, Katrin Zwirglmaier

***Correspondence:** Pia I. Scherer: pia.scherer@tum.de

1 Supplementary Material and Methods

Strains and Culture Conditions

The strains used for qPCR validations were obtained from the Culture Collection of Algae at Göttingen University in Germany (SAG) and the culture collection of the Norwegian Institute for Water Research (NIVA). They were grown under a 14/10 hrs light/dark rhythm. Fluorescent light tubes (MASTER TL5 HO 39W/865 1SL, Phillips) provided cool daylight. Cultures were grown at 20°C (*Microcystis aeruginosa* SAG14.85) or 15°C (*Dolichospermum lemmermannii* NIVA-CYA270/1, *Planktothrix agardhii* NIVA-CYA126, and *Planktothrix rubescens* SAG5.89) in BG-11 (Rippka et al., 1979).

DNA Extraction from Cultured Strains and Processing

DNA was extracted from the cells with a phenol-chloroform based method, which was modified from Fuller et al. (2003). The main modification from this protocol was that two times 2 ml of the cyanobacteria culture was spun down and the DNA was subsequently extracted from the pellet as described in the materials and methods section of the main manuscript. After extraction, DNA was carefully quantified using a NanoVue Plus spectrophotometer (GE healthcare) and diluted and aliquoted for standard curves as described in the manuscript.

Toxicity of Cultured Strains

Toxigenicity was confirmed in-house with *mcyE* primers described in Rantala et al. (2004) (data not shown). Toxicity of *M. aeruginosa* SAG14.85 was established in Lyra et al. (2001) and toxicity of *P. rubescens* SAG5.89 stain was established in Kurmayer et al. (2004). The microcystin producer stains from the NIVA culture collection were confirmed to be toxic by NIVA with microcystin ELISA.

2 Supplementary Figures

Figure S1: Separation of microcystins and nodularin-R by HPLC-PDA (standard solution, 2.5 µg/mL of each toxin, detection at 238 nm).

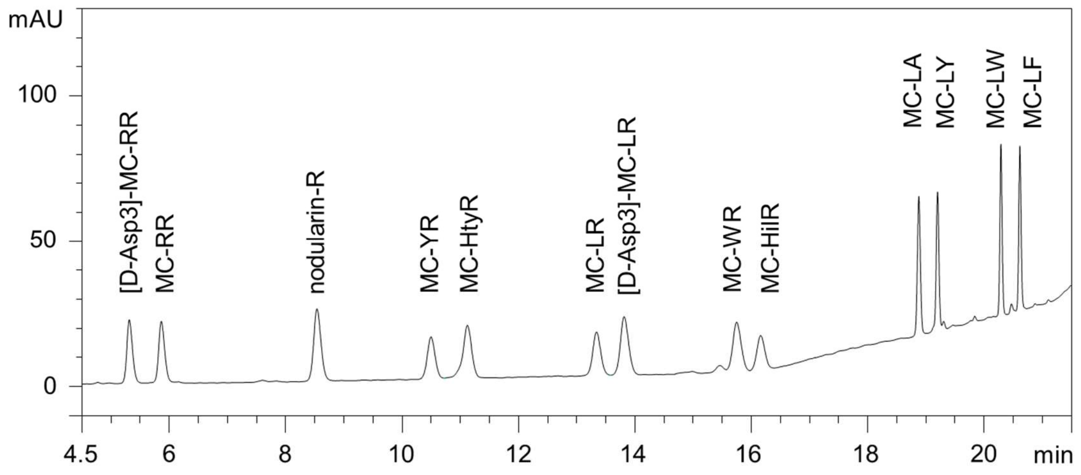

Figure S2: Rarefaction curves for each Illumina sequencing sample. Curves and sampling dates of Lake Klostersee samples in blue and Lake Bergknappweiher in green.

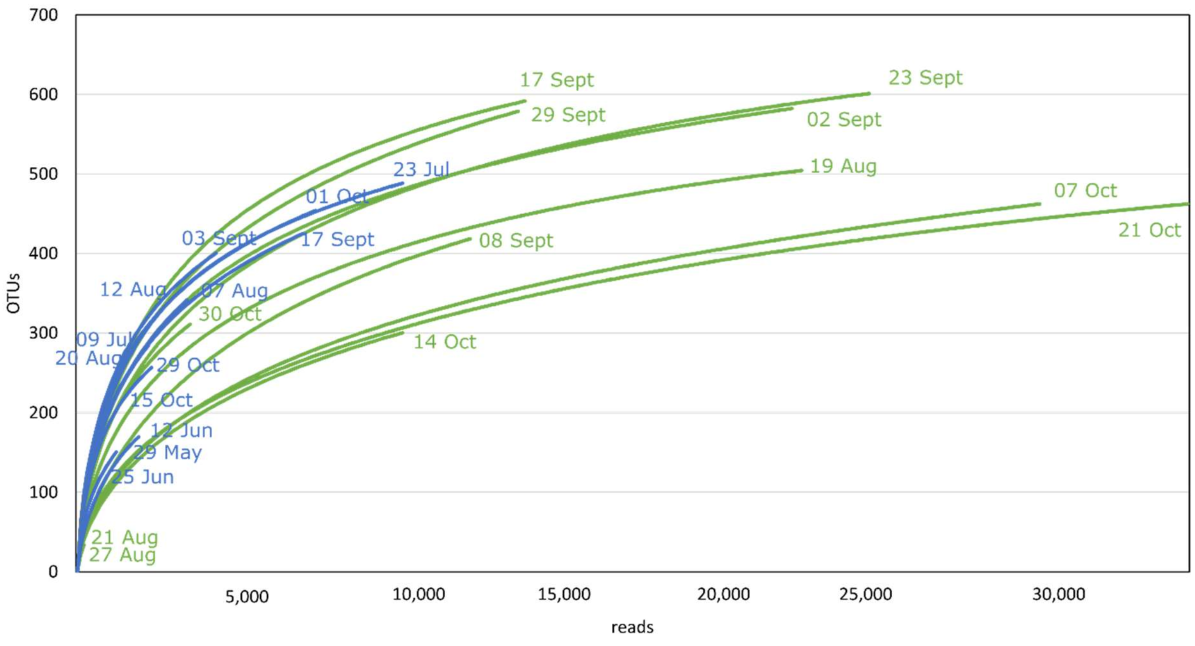

Figure S3: The qPCR assay for the detection of *Dolichospermum mcyE* (A+B) Standard curve. (A) Samples were measured in triplicates. Efficiency of standard curve is 103.4 % indicating near perfect doubling of PCR product in each cycle. R^2 value of standard curve is 0.998 indicating good linearity and low variability between technical replicates. Standard curve covers a dynamic range from 1.69×10^3 to 5.28×10^6 copies/reaction. (B) Each dilution of standard curve shows a single symmetrical melting curve peak indicating a single product. (H) qPCR products of specificity test on 2 % agarose gel. From left to right: MassRuler DNA Ladder Mix (Thermo Scientific, Waltham, US), MassRuler DNA Ladder Mix (Thermo Scientific, Waltham, US). Significant product of the right size is only present for *D. lemmermanni* as template.

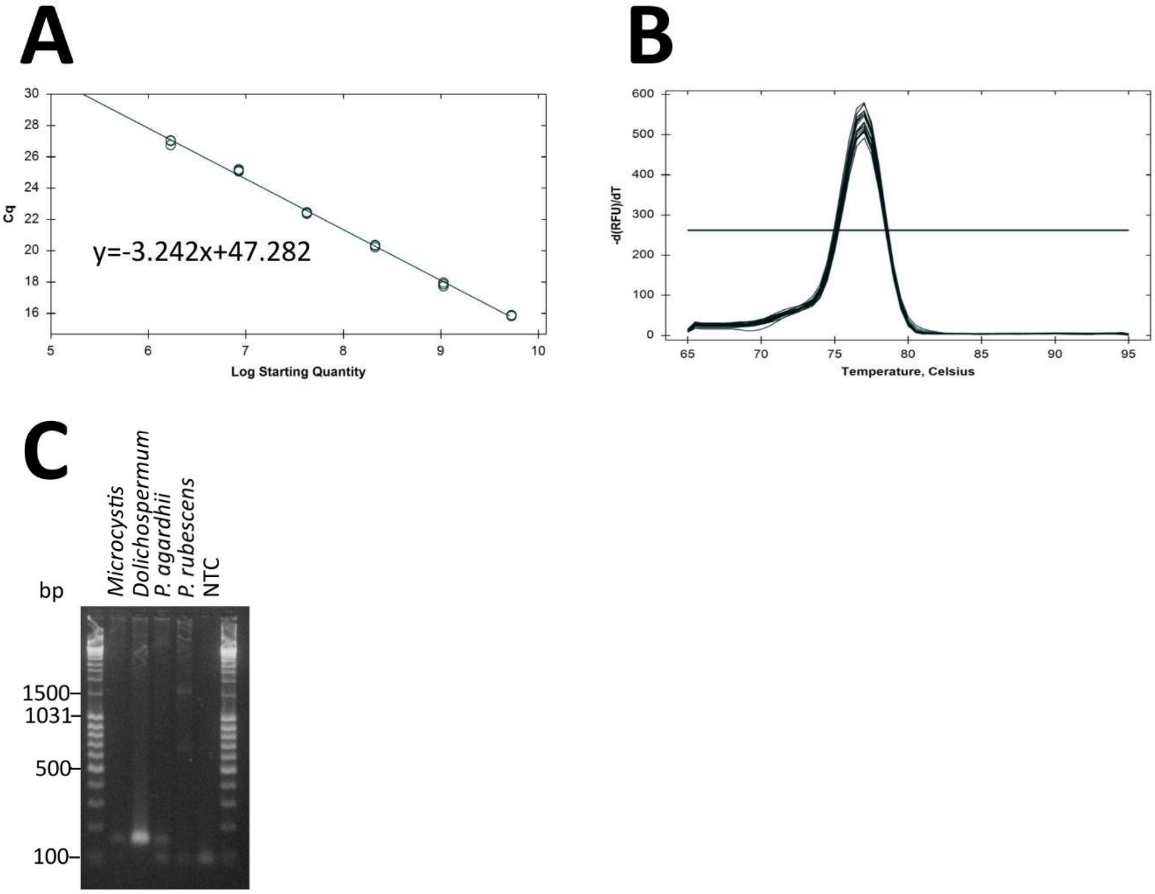

3 Supplementary Tables

Table S1: Number of sequences after quality control.

Lake	Sampling time point	Bacteria sequences	Cyanobacteria sequences	Chloroplast sequences
**Lake Klostersee**	29 May 15	1,255	381	54
	12 Jun 15	1,976	529	70
	25 Jun 15	432	56	80
	09 Jul 15	2,235	154	119
	23 Jul 15	9,071	1,392	1,167
	07 Aug 15	3,322	230	227
	12 Aug 15	2,182	245	222
	20 Aug 15	1,597	362	54
	03 Sept 15	4,211	450	220
	17 Sept 15	5,721	782	1,446
	01 Oct 15	6,775	356	813
	15 Oct 15	883	219	207
	29 Oct 15	1,853	346	592
**Lake Bergknappweiher**	19 Aug 15	21,975	8,151	648
	21 Aug 15	351	259	1
	27 Aug 15	36	15	2
	02 Sept 15	21,523	9,495	798
	08 Sept 15	12,300	8,333	36
	17 Sept 15	12,681	6,066	1,364
	23 Sept 15	23,377	12,801	1,404
	29 Sept 15	12,656	5,238	1,161
	07 Oct 15	28,402	15,809	1,650
	14 Oct 15	9,125	5,349	1,119
	21 Oct 15	29,945	14,453	4,722
	30 Oct 15	2,517	338	1,177

Table S2: Correlation of non-cyanobacteria with cyanobacteria. Pearson's correlation coefficient and p-value in brackets. Results with correlation coefficients > 0.3 or < -0.3 and p-values < 0.05 are shown in bold indicating a significant correlation.

		Microcystis	***Dolicho-spermum***	***Dolicho-spermum* OTU1**	***Synechococcus***
**Common non-cyano-bacteria**	Actinomycetales ACK-M1 OTU9	-0.06 (0.78)	**-0.73 (3.44E-5)**	**-0.49 (0.01)**	**0.64 (5.58E-4)**
	Actinomycetales ACK-M1 OTU20	-0.03 (0.88)	**-0.41 (0.04)**	-0.26 (0.21)	**0.54 (0.01)**
	Actinomycetales ACK-M1 OTU13	0.39 (0.34)	**-0.75 (1.35E-5)**	**-0.50 (0.01)**	**0.63 (8.24E-4)**
	Acidimicrobiales OTU12	-0.06 (0.78)	**-0.43 (0.03)**	**-0.46 (0.02)**	**0.49 (0.01)**
	Saprospiraceae OTU23	**0.51 (0.01)**	**-0.53 (0.01)**	**-0.43 (0.03)**	0.21 (0.32)
	Sphingomonadales OTU2	-0.21 (0.32)	**0.44 (0.03)**	**0.60 (0.001)**	-0.34 (0.09)
	Pelagibacteraceae OTU3	0.24 (0.26)	**-0.76 (1.07E-5)**	**-0.51 (0.01)**	**0.74 (2.08E-5)**
	Limnohabitans OTU5	-0.09 (0.68)	0.31 (0.13)	**0.49 (0.01)**	-0.21 (0.32)
	Ellin60607 OTU11	-0.23 (0.26)	**0.46 (0.02)**	**0.64 (5.9E-4)**	-0.35 (0.09)

Table S3: Correlation of chloroplasts with cyanobacteria and environmental factors. Pearson's correlation coefficient and in p-value brackets. Results with correlation coefficients > 0.3 or < -0.3 and p-values < 0.05 are shown in bold indicating a significant correlation.

		**Chloroplast reads (total)**	**Cryptophyta**	**Stramenopiles**	**Euglenozoa**	**Haptophyceae**
**Environ-mental para-meters**	Total phosphorus	-0.41 (0.05)	-0.25 (0.26)	-0.26 (0.24)	-0.02 (0.96)	**-0.42 (0.05)**
	$N_{inorg.}$/P	0.17 (0.46)	0.31 (0.17)	0.24 (0.28)	-0.08 (0.72)	**0.53 (0.01)**
	pH	-0.07 (0.74)	-0.14 (0.52)	-0.02 (0.93)	**0.53 (0.01)**	-0.12 (0.59)
	Water tempe-rature	**-0.54 (0.01)**	**-0.52 (0.01)**	**-0.69 (2.21E-4)**	0.29 (0.18)	0.30 (0.16)
	Secchi depth	0.13 (0.53)	-0.04 (0.86)	0.02 (0.94)	-0.14 (0.50)	**0.49 (0.02)**
	Conductivity	0.13 (0.55)	-0.07 (0.75)	0.09 (0.68)	-0.25 (0.24)	**0.51 (0.01)**
	Oxygen	0.13 (0.56)	-0.01 (0.97)	0.14 (0.51)	**0.49 (0.02)**	0.04 (0.86)
	NO_3-nitrogen	-0.24 (0.26)	0.06 (0.78)	-0.21 (0.34)	0.24 (0.26)	**-0.64 (9.46E-4)**
	Ammonium	0.41 (0.05)	0.22 (0.31)	**0.54 (0.01)**	-0.12 (0.57)	0.11 (0.61)
**Cyano-bacteria**	Cyanobacteria (total)	**-0.49 (0.01)**	-0.21 (0.32)	-0.31 (0.14)	0.10 (0.65)	**-0.64 (6.05E-4)**
	Chroococcales	-0.05 (0.82)	-0.09 (0.65)	0.05 (0.80)	-0.01 (0.97)	0.002 (0.99)
	Nostocales	**-0.46 (0.02)**	-0.21 (0.32)	-0.30 (0.14)	0.11 (0.59)	**-0.65 (4.30E-4)**
	Pseudana-baenales	**0.47 (0.02)**	0.22 (0.29)	**0.70 (9.10E-05)**	-0.10 (0.63)	0.07 (0.76)
	Synecho-coccales	-0.15 (0.46)	-0.04 (0.86)	-0.33 (0.11)	-0.08 (0.71)	0.13 (0.53)
**Toxicity para-meters**	*mcyB*	-0.20 (0.33)	-0.11 (0.61)	-0.09 (0.68)	-0.11 (0.61)	-0.26 (0.21)
	Microcystin (ELISA)	-0.12 (0.57)	-0.12 (0.57)	0.02 (0.94)	-0.09 (0.69)	-0.14 (0.50)
	Microcystin (HPLC)	0.07 (0.74)	-0.05 (0.83)	0.25 (0.25)	-0.06 (0.77)	-0.06 (0.80)

Table S4: qPCR results for each sampling date.

Lake	Sampling time point	*Microcystis mcyB* (copies/ml)	*Microcystis mcyB* standard error of the mean	*Dolichospermum mcyE* (copies/ml)
**Lake Klostersee**	29 May 15	44,200	5,600	< LOD[b]
	12 Jun 15	22,800	200	< LOD
	25 Jun 15	32,600	1,700	< LOD
	09 Jul 15	8,400	1,400	< LOD
	23 Jul 15	6,300	500	< LOD
	07 Aug 15	4,600	600	< LOD
	12 Aug 15	7,300	300	< LOD
	20 Aug 15	330,000	20,300	< LOD
	03 Sept 15	57,700	17,600	< LOD
	17 Sept 15	19,000	2,300	< LOD
	01 Oct 15	48,500	3,100	< LOD
	15 Oct 15	243,200	35,500	< LOD
	29 Oct 15	21,700	800	< LOD
**Lake Bergknappweiher**	19 Aug 15	< LOD[a]	ND	< LOD
	21 Aug 15	270,000	16,800	< LOD
	27 Aug 15	10,700	ND	< LOD
	02 Sept 15	< LOD	ND	< LOD
	08 Sept 15	606,400	74,500	< LOD
	17 Sept 15	< LOD	ND	< LOD
	23 Sept 15	< LOD	ND	< LOD
	29 Sept 15	< LOD	ND	< LOD
	07 Oct 15	147,500	1,800	< LOD
	14 Oct 15	649,900	17,800	< LOD
	21 Oct 15	92,200	11,400	< LOD
	30 Oct 15	< LOD	ND	< LOD

[a] < 612 copies/mL for *Microcystis mcyB;* [b] < 3,380 copies/mL for *Dolichospermum mcyE*

Table S5: Chemical and physical water parameters for each sampling date, ND = value not determined, LOD = limit of detection.

Lake	Sampling time point	Microcystin ELISA (µg/L)	Microcystin HPLC (µg/L)	Visible surface bloom	Total phosphorus (µg/L)	$N_{inorg.}/P$ (stoichiometric)	pH	Water temperature (°C)	Secchi depth (m)	Conductivity (µS/cm²)	Oxygen (mg/L)	NO_3-nitrogen (mg/L)	Ammonium (mg/L)
**Lake Klostersee**	29 May 15	ND	ND	yes	ND	ND	8.09	19.0	1.00	431	9.16	ND	ND
	12 Jun 15	1.0	< LOD[b]	yes	80	12	8.36	21.3	0.75	423	10.52	0.29	0.19
	25 Jun 15	1.0	< LOD	yes	57	18	8.40	19.5	0.75	415	12.22	0.35	0.15
	09 Jul 15	< LOD[a]	< LOD	yes	60	22	8.06	24.3	0.80	414	6.71	0.42	0.23
	23 Jul 15	< LOD	< LOD	yes	39	28	8.07	26.1	1.50	405	7.33	0.36	0.16
	07 Aug 15	< LOD	< LOD	no	47	21	8.52	24.9	0.75	399	10.13	0.30	0.17
	12 Aug 15	< LOD	< LOD	no	43	20	8.22	25.8	0.75	390	9.46	0.40	0.00
	20 Aug 15	1.2	< LOD	yes	160	5	7.85	21.6	0.75	398	ND	0.39	0.00
	03 Sept 15	< LOD	< LOD	no	62	11	7.89	21.7	0.60	380	6.97	0.32	0.00
	17 Sept 15	< LOD	< LOD	no	52	18	8.67	18.5	0.40	377	12.33	0.34	0.12
	01 Oct 15	< LOD	< LOD	yes	67	18	8.32	13.6	0.75	392	9.83	0.37	0.23
	15 Oct 15	5.0	5.5	yes	230	5	8.16	11.4	0.70	396	8.48	0.29	0.24
	29 Oct 15	< LOD	< LOD	no	68	25	7.95	10.1	0.90	410	8.05	0.40	0.48
**Lake Bergknappweiher**	19 Aug 15	< LOD	ND	no	282	ND	ND	ND	ND	ND	ND	ND	ND
	21 Aug 15	1.4	< LOD	yes	1,034	2	8.51	20.7	0.15	257	10.78	1.08	0.06
	27 Aug 15	< LOD	< LOD	yes	181	13	9.13	25.1	0.45	243	14.85	1.00	0.06
	02 Sept 15	< LOD	< LOD	yes	155	16	8.20	21.9	0.50	256	5.20	1.09	0.06
	08 Sept 15	6.7	3.4	yes	834	3	8.08	15.4	0.40	255	7.79	1.07	0.06
	17 Sept 15	< LOD	< LOD	no	162	13	8.54	18.2	0.40	253	8.82	0.92	0.06
	23 Sept 15	< LOD	< LOD	no	204	10	8.70	15.7	0.50	253	10.21	0.83	0.05
	29 Sept 15	< LOD	< LOD	no	132	15	8.49	14.8	0.40	253	11.80	0.85	0.07
	07 Oct 15	< LOD	< LOD	no	110	17	8.57	14.2	0.40	256	9.57	0.83	0.05
	14 Oct 15	< LOD	< LOD	yes	174	12	8.27	10.5	0.40	259	9.04	0.91	0.05
	21 Oct 15	< LOD	< LOD	yes	ND	ND	8.70	10.2	0.60	259	12.98	0.83	0.05
	30 Oct 15	< LOD	< LOD	no	94	22	8.15	9.7	0.50	270	9.81	0.87	0.06

[a] < 1.0 µg/L; [b] none of the target analytes was detected (< 1.0 µg/L)

Table S6: Correlation of water quality parameters. Pearson's correlation coefficient and p-value in brackets. Results with correlation coefficients > 0.3 or < -0.3 and p-values < 0.05 are shown in bold indicating a significant correlation.

		mcyB	**Total phos-phorus**	**$N_{inorg.}$/P**	**pH**	**Water tempe-rature**	**Secchi depth**	**Con-ductivity**	**Oxygen**	**NO_3-nitrogen**	**Ammo-nium**
**Toxicity para-meters**	*mcyB*		**0.56 (0.01)**	**-0.61 (0.003)**	-0.23 (0.28)	-0.32 (0.13)	-0.31 (0.14)	-0.26 (0.21)	-0.14 (0.5)	0.31 (0.15)	-0.22 (0.32)
	Micro-cystin (ELISA)	**0.61 (1.53E-3)**	**0.59 (0.003)**	**-0.63 (0.002)**	-0.25 (0.25)	-0.19 (0.40)	-0.12 (0.59)	-0.01 (0.96)	-0.16 (0.46)	0.08 (0.71)	0.04 (0.84)
	Micro-cystin (HPLC)	**0.44 (0.04)**	0.35 (0.11)	**-0.49 (0.02)**	-0.21 (0.34)	-0.28 (0.19)	-0.03 (0.89)	0.05 (0.84)	-0.20 (0.37)	-0.04 (0.84)	0.16 (0.46)

Table S7: Correlation of cyanobacteria with environmental factors. Pearson's correlation coefficient and p-value in brackets. Results with correlation coefficients > 0.3 or < -0.3 and p-values < 0.05 are shown in bold indicating a significant correlation.

		Microcystis	***Dolicho-spermum***	**Chroococcales OTU594**	**Gomphos-phaeriaceae**	***Snowella* OTU65**	**Pseudana-baenaceae**	***Pseudana-baena* OTU71**	***Synecho-coccus***
**Environ-mental para-meters**	Total phosphorus	**0.44 (0.03)**	**-0.45 (0.03)**	0.30 (0.16)	0.07 (0.74)	**0.86 (1.48E-7)**	-0.06 (0.80)	-0.05 (0.81)	**0.43 (0.04)**
	$N_{inorg.}/P$	-0.39 (0.07)	0.35 (0.11)	-0.29 (0.19)	-0.19 (0.40)	**-0.54 (0.01)**	-0.04 (0.86)	0.19 (0.41)	-0.32 (0.14)
	pH	**0.52 (0.01)**	**-0.45 (0.03)**	**0.44 (0.03)**	0.35 (0.09)	0.11 (0.58)	0.11 (0.60)	-0.002 (0.99)	0.26 (0.22)
	Water temperature	-0.33 (0.11)	0.16 (0.44)	-0.09 (0.67)	-0.31 (0.14)	0.16 (0.45)	-0.39 (0.06)	**-0.48 (0.02)**	0.24 (0.26)
	Secchi depth	-0.32 (0.13)	**0.65 (6.56E-4)**	-0.27 (0.21)	-0.27 (0.20)	**-0.46 (0.02)**	-0.35 (0.10)	-0.17 (0.43)	**-0.47 (0.02)**
	Conductivity	**-0.51 (0.01)**	**0.91 (1.19E-9)**	**-0.50 (0.01)**	**-0.50 (0.01)**	-0.35 (0.09)	**-0.44 (0.03)**	-0.32 (0.13)	**-0.59 (0.003)**
	Oxygen	**0.47 (0.02)**	-0.09 (0.68)	**0.40 (0.06)**	0.15 (0.49)	0.01 (0.98)	0.002 (0.99)	0.12 (0.58)	-0.12 (0.58)
	NO_3-nitrogen	**0.44 (0.04)**	**-0.93 (1.68E-10)**	**0.50 (0.01)**	0.38 (0.07)	**0.48 (0.02)**	0.34 (0.12)	0.28 (0.20)	**0.69 (2.76E-4)**
	Ammonium	-0.21 (0.34)	**0.43 (0.04)**	-0.25 (0.25)	-0.26 (0.23)	-0.20 (0.35)	-0.26 (0.23)	-0.14 (0.52)	-0.27 (0.22)

4 References

Fuller, N.J., Marie, D., Partensky, F., Vaulot, D., Post, A.F., and Scanlan, D.J. (2003). Clade-specific 16S ribosomal DNA oligonucleotides reveal the predominance of a single marine Synechococcus clade throughout a stratified water column in the Red Sea. *Appl Environ Microbiol* 69**,** 2430-2443. doi: 10.1128/AEM.69.5.2430-2443.2003

Kurmayer, R., Christiansen, G., Fastner, J., and Börner, T. (2004). Abundance of active and inactive microcystin genotypes in populations of the toxic cyanobacterium *Planktothrix* spp. *Environ Microbiol* 6**,** 831-841. doi: 10.1111/j.1462-2920.2004.00626.x

Lyra, C., Suomalainen, S., Gugger, M., Vezie, C., Sundman, P., Paulin, L., and Sivonen, K. (2001). Molecular characterization of planktic cyanobacteria of *Anabaena*, *Aphanizomenon*, *Microcystis* and *Planktothrix* genera. *Int J Syst Evol Microbiol* 51**,** 513-526. doi: 10.1099/00207713-51-2-513

Rantala, A., Fewer, D.P., Hisbergues, M., Rouhiainen, L., Vaitomaa, J., Borner, T., and Sivonen, K. (2004). Phylogenetic evidence for the early evolution of microcystin synthesis. *Proc Natl Acad Sci U S A* 101**,** 568-573. doi: 10.1073/pnas.0304489101

Rippka, R., Deruelles, J., Waterbury, J.B., Herdman, M., and Stanier, R.Y. (1979). Generic assignments, strain histories and properties of pure cultures of cyanobacteria. *J Gen Microbiol* 111**,** 1-61. doi: 10.1099/00221287-111-1-1
